# Research on the Biofilm Formation of *Staphylococcus aureus* after Cold Stress

**DOI:** 10.3390/microorganisms9071534

**Published:** 2021-07-19

**Authors:** Jiaju Qiao, Liping Zheng, Zhaoxin Lu, Fanqiang Meng, Xiaomei Bie

**Affiliations:** College of Food Science and Technology, Nanjing Agricultural University, Nanjing 210095, China; 2016208008@njau.edu.cn (J.Q.); 2018108004@njau.edu.cn (L.Z.); fmb@njau.edu.cn (Z.L.); mfq@njau.edu.cn (F.M.)

**Keywords:** foodborne pathogens, cold stress, biofilm, polysaccharide intercellular adhesion

## Abstract

*Staphylococcus aureus* is a common food pathogen and has a strong tolerance to environmental stress. Here, the biofilm formation of *S. aureus* strains after cold stress for 24 weeks were investigated. It was found that the biofilm formation of *S. aureus* CICC 21600, CICC 22942, W1, W3, and C1 cells was enhanced after cold stress for 20 weeks. What is more, the mRNA levels of the *clfA*, *icaA*, *icaB*, *icaC* or *icaD* genes in these strains were increased for >2-fold. The increased gene transcription levels were consistent with the increase in the polysaccharide content in the biofilm matrix of these *S. aureus* strains after cold stress. Meanwhile, hydrophobicity and the adhesion proteins also played a role in the formation of biofilms. The biofilm of *S. aureus* cells can be effectively degraded by snailase and proteinase K (125 µg/mL + 20 µg/mL) mixture. In summary, *S. aureus* frozen at −20 °C for 12 to 20 weeks is still a potential hazard. Food factory equipment should be cleaned in a timely manner to avoid outbreaks of foodborne pathogenic bacteria due to contamination.

## 1. Introduction

*Staphylococcus aureus* is a well-known foodborne pathogen, and it is one of the main microbes that causes disease outbreaks related to food consumption [1]. It has been found in various foods, such as poultry [2], meat [3], ice cream [4], milchigs [1], sushi, sashimi [5], dumplings, and rice balls [6]. There are approximately 241,000 cases of foodborne diseases in the United States caused by *S. aureus* every year [7]. Approximately 20–25% of foodborne outbreaks are caused by *S. aureus* in China [8].

The formation of biofilm promotes the survival of many bacteria in the natural environment [9]. Biofilms are heterogeneous mixtures of a secreted matrix and bacteria. This kind of matrix is also called extracellular polymeric substances [9]. Moreover, the composition of the biofilm matrix varies with strain specificity, but can usually contain host factors, polysaccharides, proteins, and extracellular DNA (eDNA) [10]. It is well known that the main component of the biofilm matrix of *S. aureus* is polysaccharide intercellular adhesion (PIA). The synthesis and accumulation of PIA are mediated by *ica* locus, which includes four genes: *icaA*, *icaD*, *icaB*, and *icaC* [11]. The transcription of the *icaADBC* gene is affected by a number of regulatory and environmental factors [12]. Additionally, various adhesion proteins have been documented that promote the formation of biofilms in *ica*-independent *S. aureus* [13]. These proteins included the surface proteins SasG, Protein A, the clumping factor B (ClfB), the fibronectin-binding proteins (FnBPA). In addition, the hydrophobic surface proteins can induce nonspecific electrostatic or hydrophobic interactions, thus promoting the primary adhesion of strains on abiotic surfaces [14].

*S. aureus* was found in a variety of environments, indicating it is a highly adaptable organism [15]. In the process of food production, processing, storage, distribution, or preparation, foodborne pathogens may be subjected to various stresses or damage. Usually, these pathogens can perceive differences in the environment and respond by altering the transcription levels of mRNA [16]. Low temperature is the main method used to reduce the microorganisms in the food industry [17]. However, there are still few studies on the biofilm-formation of *S. aureus* cells after cold stress. Therefore, eight strains of *S. aureus* after being cold-stressed for 20 weeks were used to study the biofilm-formation. The frozen bacterial suspension was under the normal temperature of nature defrosting. *S. aureus* strains after cold stress were diluted 10-fold and spread on tryptic soy broth agar plate to calculate colony forming units [18]. The survival rate of *S. aureus* cells after cold-stressed at −20 °C for 20 weeks was higher than 65%. The surviving population after defrosting was enumerated in all experiments. After cold at −20 °C, *S. aureus* strains could still grow, develop resistance to antibiotics, and form biofilms. In the early stage of the experiment, it was found that the Y410F mutation of topoisomerase IV subunit-GlrA in *S. aureus* CICC 10201 cells after cold stress. The *S. aureus* W3 strains that survived the cold stress were genetically identical to the parent strains. This suggested that the mutation was not necessarily related to the survival of the strain. Furthermore, the purposes of this paper were as follows: (1) to evaluate the changes in biofilm formation of *S. aureus* cells with cold stress; (2) to discuss the effects of cold stress on the transcription of the genes associated with biofilm-formation in *S. aureus*; and (3) to study the effects of cold stress on the content of matrix in biofilms of *S. aureus* cells. To understand the rules of biofilm formation in *S. aureus* cells with cold stress, pathogenic bacteria should be controlled more effectively.

## 2. Materials and Methods

### 2.1. Materials

*S. aureus* CICC 10201, CICC 21600, CICC 22942, and CICC 10788 were purchased from the Chinese Center of Industrial Culture Collection. *S. aureus* W3, W1, C1 and C4 were isolated from cold dumplings and cold dishes in our laboratory. *TransZol* Up was obtained from TransGen (Beijing, China), the cDNA synthesis kit was purchased from ABM (Nanjing, China), and the qPCR SYBR Green Master Mix was purchased from Yeasen (Shanghai, China).

### 2.2. Biofilm Formation of S. aureus Cells with Cold Stress

Bacterial biofilms were cultured in nutrient broth medium and measured with the crystal violet staining method [19,20]. *S. aureus* CICC 10201, CICC 21600, CICC 22942, CICC 10788, W3, W1, C1, and C4 strains without cold stress were cultured as controls. The *S. aureus* cells were cultured to the mid-log phase (10^9^ CFU/mL) with tryptic soy broth (TSB). 1.6 mL volume of the bacterial suspension (stored in TSB) was placed in a freezing tube and placed in the −20 °C refrigerator. Strains that underwent cold stress at −20 °C for 4 weeks, 8 weeks, 12 weeks, 16 weeks, 20 weeks, and 24 weeks were used as test samples. The frozen bacterial suspension was under the normal temperature of nature defrosting. Then the concentration of bacterial suspension was adjusted to an OD_595_ of 0.5 (10^8^ CFU/mL) by Nutrient Broth medium. Every sample (10^6^ CFU/mL) was added to 96-well microtiter plates at 200 µL per well, 8 wells were repeated. After plates were incubated at 37 °C for 48 h, the culture medium was discarded. The biofilms were washed three times with sterile water. After drying, 200 µL crystal violet dye was then added to each well and incubated for 30 min. The biofilms were then rinsed with sterile water to remove the dye. After drying, 200 µL of 95% ethanol was added to every well and incubated for 20 mins at room temperature. Finally, OD_595_ values were measured to determine the formation of biofilms using a microplate reader.

### 2.3. The Growth of S. aureus Biofilm Cells after Cold Stress

*S. aureus* CICC 10201, C4, W1, W3, CICC 21600, CICC 22942, C1, and CICC 10788 strains after cold stress at −20 °C for 20 weeks were cultured as the test group. Strains without cold stress was as control group. The treatment of *S. aureus* strains after cold stress was similar to Section 2.2. Every sample (10^6^ CFU/mL) was added to 6-well microtiter plates at 5 mL per well, 3 wells were repeated. After plates were incubated at 37 °C for 48 h, the culture medium was discarded and the biofilm cells were resuspended in TSB. Then the concentration of the *S. aureus* biofilm cells was adjusted to 10^6^ CFU/mL. The bacteria cells was cultured with 180 rpm at 37 °C for 48 h. Finally, OD_600_ values were measured per 30 min to determine the growth of the bacteria cells using a microplate reader.

### 2.4. Relative Transcription Levels of Biofilm-Associated Genes

#### 2.4.1. RNA Extraction

*S. aureus* CICC 10201, CICC 21600, CICC 22942, CICC 10788, W3, W1, C1 and C4 strains under cold stress at −20 °C for 20 weeks were cultured as the test group. The treatment of *S. aureus* strains after cold stress was similar to Section 2.2. *S. aureus* cells without cold stress were the control group. The concentration of the *S. aureus* cells was adjusted to 10^6^ CFU/mL. The strains were then inoculated in NB medium and biofilm statically developed at 37 °C for 48 h. The supernatant was discarded and the biofilm cells were resuspended in PBS. First, 1 mL of bacterial suspension of the *S. aureus* strain was centrifuged (12,000× *g*, 2 min) to collect cells. Second, 1 mL of *TransZol* Up was added to the cell lysate for 5 min. The mixture was incubated at 25 °C for 5 min after 200 µL of trichloromethane was added. Third, 400 µL of supernatant from the mixture was transferred to a new centrifuge tube after centrifugation (12,000× *g*, 10 min). 400 µL of isopropyl alcohol was added and incubated for 15 min at room temperature. After centrifugation at 12,000× *g* for 10 min, the supernatant liquid was discarded. One milliliter of precooled 75% ethanol was added for washing, with further centrifugation at 12,000 × *g* for 5 min. The wash step was then repeated. After air-drying, 40 µL of RNA dissolving solution was added and heated for 10 min at 65 °C prior to cDNA preparation [21].

#### 2.4.2. Reverse Transcription

RNA was transcribed into cDNA using a cDNA synthesis kit. The cDNA was stored at −80 °C.

#### 2.4.3. RT-PCR

Using cDNA as a template, RT-PCR was performed by mixing 10 μL of qPCR SYBR Green Master Mix (ROX), 0.4 μL of each primer (0.2 μmol/L), 1 μL of cDNA template, and 8.2 μL of RNase-free ddH_2_O. All experiments were performed in triplicate, and the procedure were listed in Table 1 [22]. Melting analysis was performed by keeping the denaturation reaction at 95 °C for 15 s, and hybridization at 60 °C for 1 min, followed by heating from 60 °C to 95 °C at 0.3 °C/s [23,24]. The relative transcription of target genes was described by 2^−ΔΔC^^T^, while *16S rRNA* gene was assayed as an internal control. Strains cultured at 37 °C were used as controls, and the relative transcription of biofilm-related genes (*icaA*, *icaB*, *icaC*, *icaD*, *cflA*, *fnbpA*, *spa* and *sasG*) was determined for *S. aureus* CICC 10201, CICC 21600, CICC 22942, CICC 10788, W3, W1, C1 or C4 after cold stress. Calculations were as follows: ΔC_T_ (test or control) = C_T_ (target gene)‒C_T_ (internal control), ΔΔC_T_ = ΔC_T_ (test)‒ΔC_T_ (control). The primers used in this investigation are described in Table 2. The efficiencies of each primer pair was calculated [25,26], and presented in Table 2.

### 2.5. Detection of Extracellular Polysaccharides in Biofilm Matrix

*S. aureus* CICC 10201, CICC 21600, CICC 22942, CICC 10788, W3, W1, C1 or C4 cells with or without cold stress were cultured for 48 h to form biofilms. After washing with PBS, then the biofilm matrix was collected with a cell scraper and centrifuged at 12,000× *g* for 20 min. The supernatant was sterilized with a 0.22 μm filter membrane. Then, the filtrate was purified overnight using a 3500 Da dialysis membrane. The purified biofilm matrix was lyophilized and concentrated and then resuspended in sterile water [28].

To determine polysaccharides, 25 μg/mL, 50 μg/mL, 100 μg/mL, 150 μg/mL and 200 μg/mL glucose standard solutions were prepared. One hundred microliters of glucose solution was mixed with 100 μL of 5% phenol solution and 200 μL of 98% sulfuric acid, then incubated in a water bath at 90 °C for 30 min. The absorbance was measured at 490 nm by ultraviolet spectrophotometry. The linear regression equation of the standard curve of the phenol-sulfuric acid method was as follows: Y = 0.0113X + 0.0254, R² = 0.9997. The X: the concentration of polysaccharide in the biofilm matrix. Y: the absorbance was measured at 490 nm [28].

### 2.6. Evaluation of Protein in Biofilm Matrix

The protein contents were determined by BCA protein assay kits. Twenty microliter volumes of 0 μg/mL, 1 μg/mL, 2 μg/mL, 8 μg/mL, and 12 μg/mL protein standard solutions were prepared, and 100 μL of BCA working solution was added. Then, the mixture was incubated in a 60 °C water bath for 30 min. The absorbance was measured at 562 nm according to ultraviolet spectrophotometry, and the standard curve was as follows: y = 0.2734x + 0.1799, R² = 0.9995. (X: the concentration of protein in biofilm matrix. Y: the absorbance was measured at 562 nm). The purified biofilm matrix (mentioned in 2.4) of *S. aureus* CICC 10201, CICC 21600, CICC 22942, CICC 10788, W3, W1, C1 or C4 with cold stress was mixed with BCA working solution. *S. aureus* cells without stress were used as the control group. The protein concentration was calculated according to the OD_562_ value [28].

### 2.7. Hydrophobicity of S. aureus Strains after Cold Stress

The cell-surface hydrophobicity of *S. aureus* CICC 10201, CICC 21600, CICC 22942, CICC 10788, W3, W1, C1 or C4 with or without cold stress was detected using the bacterial adhesion to hydrocarbons (BATH) [29,30]. *S. aureus* strains with or without cold stress were cultured at 37 °C for 48 h, washed with PBS and resuspended in PBS (10 mM pH 7.4). The cell concentration of the resuspended bacterial solution was adjusted to 0.8 ± 0.05 at OD_540_, which was recorded as OD_0_. Then, 1 mL of xylene was mixed with 4 mL of cell suspension and vortexed vigorously for 120 s. The water phase and the organic phase of the samples were separated after incubation at room temperature for 30 min. The lower water phase sample was removed, and the OD value at 540 nm was recorded as OD_1_. The formula for calculating the hydrophobicity of *S. aureus* strains after cold stress was as follows: CSH (%) = [(OD_0_–OD_1_)/OD_0_] × 100%. 

### 2.8. Antibiotics and Enzymes to Remove S. aureus Biofilm

Bacterial biofilm formation by *S. aureus* W3 in nutrient broth medium was measured using the 96-well plate crystal violet staining method [19,20]. Briefly, every sample (10^6^ CFU/mL) was added to a 96-well microtiter plates at 200 µL per well and repeated in 6 wells. After incubation at 37 °C for 24 h, the culture medium was discarded. Then various concentrations of norfloxacin, levofloxacin, ciprofloxacin (1, 2, 4, 8, 16, and 32 μg/mL), proteinase K (500, 100, 20, 4, 0.8, or 0.16 μg/mL) and snailase (125, 25, 5, 1, or 0.5 μg/mL) were added into the wells respectively, incubated for 1 h. Then, these solutions were discarded and biofilms were washed twice by water. Finally, OD_595_ values of crystal violet retention were measured to determine the extent of biofilm removal.

### 2.9. The Proteinase K, Snailase, or 84 Disinfectant Degraded S. aureus Biofilm

Bacterial biofilm formation by *S. aureus* CICC 10201, CICC 21600, CICC 22942, CICC 10788, W3, W1, C1, or C4 cells was measured using the 96-well plate crystal violet staining method [19,20]. Briefly, every sample (10^6^ CFU/mL) was added to 96-well microtiter plates at 200 µL per well and repeated in 6 wells. After incubation at 37 °C for 24 h, the culture medium was discarded. Then snailase and norfloxacin mixture (125 µg/mL + 16 µg/mL), snailase and proteinase K mixture (125 µg/mL + 20 µg/mL), NOR and proteinase K mixture (16 µg/mL + 20 µg/mL), snailase (125 µg/mL) and 84 disinfectant (disinfectant with sodium hypochlorite, 1%) were added into the wells respectively, incubated for 1 h. Then, these solutions were discarded, and biofilms were washed twice by water. Finally, OD_595_ values of crystal violet retention were measured to determine the extent of biofilm removal.

### 2.10. Statistical Analysis

Statistical differences were evaluated using IBM SPSS Statistics 20 (New York, NY, USA), and all experiments were repeated at least three times. One-way ANOVA was used in this study for every Figure and means were compared using Tukey’s multiple range tests. The values were means ± standard deviations. A probability value of < 0.05 was considered significant. “*” indicates *p* < 0.05, “**” indicates *p* < 0.01, compared to the control group. The results were plotted using Origin 8.5 software.

## 3. Results

### 3.1. Biofilm Formation of Staphylococcus aureus Strains after Cold Stress

**Figure 1:**
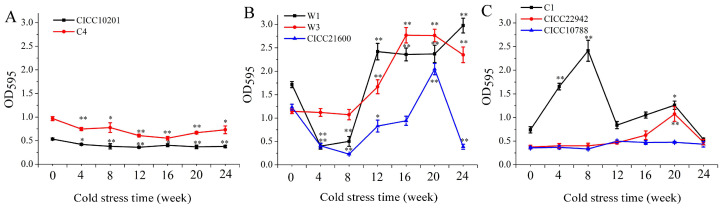
Biofilm formation of *S. aureus* cells after cold stress at −20 °C. Note: (**A**): Biofilm formation of *S. aureus* CICC 10201 and C4. (**B**): Biofilm formation of *S. aureus* W1, W3 and CICC 21600. (**C**): Biofilm formation of *S. aureus* C1, CICC 22942 and CICC 10788. Strains without cold stress were used as control samples, and strains after cold stress at −20 °C for 4 weeks, 8 weeks, 12 weeks, 16 weeks, 20 weeks, or 24 weeks were used as the treatment groups. **: Compared with the control groups, the tested sample were significantly different (*p* < 0.01), *: Compared with the control group, the treated sample were different (*p* < 0.05). The values were means ± standard deviations (SD, *n* = 8).

To detect the effect of cold stress on biofilm formation, eight strains of *S. aureus* were cold stressed at −20 °C for several weeks. The *S. aureus* cells without cold stress were used as the control group. Compared with the control group, the biofilm formation of *S. aureus* CICC 10201 (the absorbance decreased from 0.53 ± 0.02 to 0.36 ± 0.01 at OD_595_) and C4 (from 0.97 ± 0.04 to 0.55 ± 0.04) decreased significantly after cold stress at −20 °C for 4–24 weeks (*p* < 0.05, Figure 1A). However, the biofilm formation trend of cold-resistant *S. aureus* W1 (the absorbance increased from 1.71 ± 0.06 to 2.98 ± 0.15 at OD_595_) or W3 (from 1.14 ± 0.05 to 2.77 ± 0.13) was similarly increased after cold stress for 12 weeks, 16 weeks, 20 weeks, and 24 weeks (*p* < 0.01, Figure 1B). The biofilm formation of the *S. aureus* CICC 21600 strain after cold stress for 4–8 weeks (the absorbance decreased from 1.23 ± 0.06 to 0.23 ± 0.01 at OD_595_) was obviously decreased (*p* < 0.01, Figure 1B), similar to that of *S. aureus* W1 after cold stress. Meanwhile, the biofilm formation of *S. aureus* CICC 21600 cells after cold stress for 12–20 weeks was upregulated gradually. Specifically, the biofilm formation of *S. aureus* CICC 21600 cells increased significantly after 20 weeks of cold stress (the absorbance increased from 1.23 ± 0.06 to 2.05 ± 0.12 at OD_595_, *p* < 0.01). Biofilm formation of the *S. aureus* CICC 21600 strain exhibited a downward trend with cold stress for 24 weeks (Figure 1B).

Compared with the control group, the biofilm formation of *S. aureus* C1 cells was upregulated after 4 weeks of cold stress at −20 °C (Figure 1C), and the difference was significant at 4 weeks, 8 weeks and 20 weeks (The absorbance increased from 0.74 ± 0.06 to 1.26 ± 0.08 at OD_595_, *p* < 0.05). Moreover, the biofilm formation of *S. aureus* CICC 22942 cells after cold stress for 4 weeks, 8 weeks, 12 weeks, 16 weeks and 20 weeks increased gradually. The difference was remarkable in the *S. aureus* CICC 22942 cells after cold stress for 20 weeks (The absorbance increased from 0.37 ± 0.03 to 1.07 ± 0.15 at OD_595_, *p* < 0.01). However, when *S. aureus* CICC 10788 was exposed to cold stress of −20 °C for 12 weeks, 16 weeks, 20 weeks, or 24 weeks, the biofilm formation of *S. aureus* cells was increased, but the difference was not obvious.

In summary, the biofilm formation of *S. aureus* CICC 21600, CICC 22942, W1, W3, and C1 cells were all increased significantly (*p* < 0.01, *p* < 0.05) after cold stress for 20 weeks at −20 °C.

### 3.2. The Growth of S. aureus Biofilm Cells after Cold Stress

**Figure 2:**
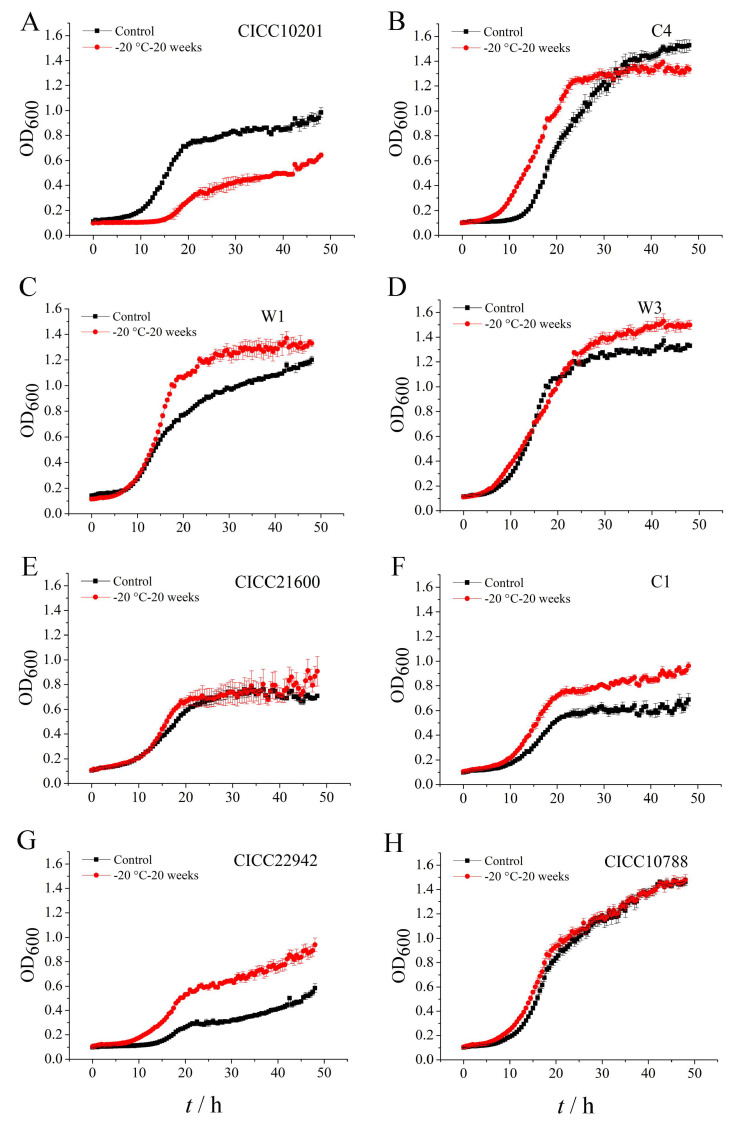
The growth of *S. aureus* biofilm cells after cold stress at −20 °C for 20 weeks. Note: The growth curve of biofilm cells of *S. aureus* CICC 10201 (**A**), *S. aureus* C4 (**B**), *S. aureus* W1(**C**), *S. aureus* W3 (**D**), *S. aureus* CICC 21600 (**E**), *S. aureus* C1 (**F**), *S. aureus* CICC 22942 (**G**) and *S. aureus* CICC 10788 (**H**) was represented by the OD value at 600 nm. The biofilm cells of *S. aureus* strains without cold stress were used as control groups. The biofilm cells of *S. aureus* strains after cold stress at −20 °C for 20 weeks were used as treatment groups.

In order to investigate the viability of *S. aureus* biofilm cells after cold stress, we detected the growth of biofilm bacteria after cold stress at −20 ℃ for 20 weeks. Biofilm bacteria without cold stress were used as the control group. Compared with the control group, the logarithmic phase growth of *S. aureus* W1, C1, or CICC 22942 biofilm cells after cold stress at −20 ℃ for 20 weeks was longer. The total count of *S. aureus* W1, W3, C1, or CICC 22942 biofilm cells with cold stress accumulated more in logarithmic phase or stable phase (Figure 2C,D,F,G). We suggested the potential contamination of *S. aureus* biofilm bacteria increased after cold stress at −20 ℃ for 20 weeks. 

Obviously, compared with the control group, the logarithmic phase growth of *S. aureus* CICC 10201 biofilm cells after cold stress at −20 ℃ for 20 weeks was slow. The total count of *S. aureus* CICC 10201 or C1 biofilm cells with cold stress accumulated less in the stable phase (Figure 2A,B). Moreover, compared with the control group, the growth of *S. aureus* CICC 21600 or CICC 10788 biofilm cells after cold stress was not changed (Figure 2E,H).

### 3.3. Transcription of Genes in Staphylococcus aureus Related to Biofilm Formation

**Figure 3:**
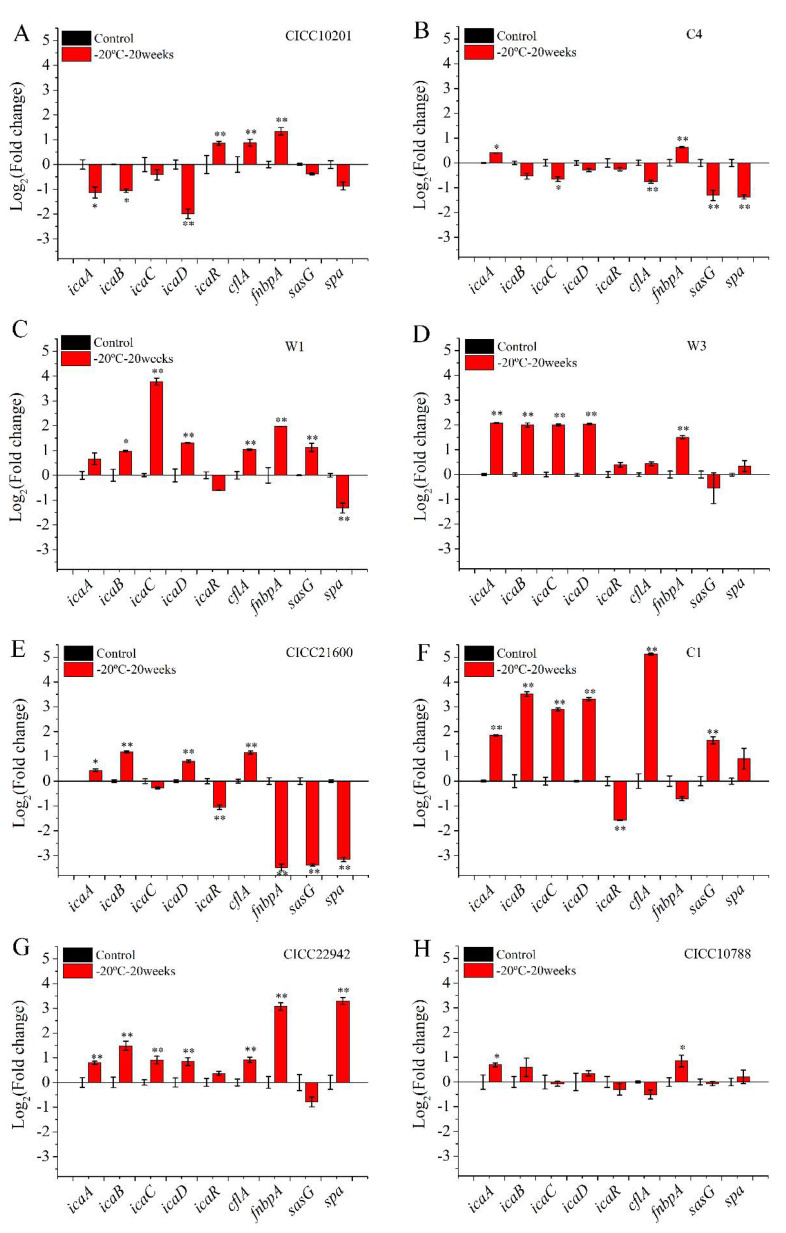
The transcription levels of genes in *S. aureus* biofilm cells with cold stress. Note: The transcription levels of genes in biofilm cells of *S. aureus* CICC 10201 (**A**), *S. aureus* C4 (**B**), *S. aureus* W1 (**C**), *S. aureus* W3 (**D**), *S. aureus* CICC 21600 (**E**), *S. aureus* C1 (**F**), *S. aureus* CICC 22942(**G**), *S. aureus* CICC 10788 (**H**) was measured by Real-Time PCR. *S. aureus* without cold stress was used as the control group. The gene transcription levels in biofilm cells of *S. aureus* after cold stress for 20 weeks at −20 °C were tested. The polysaccharide intercellular adhesin (PIA) is mainly regulated by *icaABCDR* genes gene. The adhesion proteins included *sasG*, *cflA*, *spa*, or *fnbpA* genes. **: Compared with the control group, the tested sample was significantly different (*p* < 0.01), *: Compared with the control group, the treated sample was different (*p* < 0.05). The values were means ± SD (*n* = 3).

To investigate the effect of cold stress on biofilm formation, gene transcription was tested in *S. aureus* after cold stress for 20 weeks at −20 °C. *S. aureus* cells without cold stress was used as a control sample, and that after cold stress was used as the treatment group (Figure 3). Compared with the control group, the transcription level of the *icaA*, *icaB*, *icaC*, or *icaD* gene was increased by over 2-fold (*p* < 0.01) in biofilms of *S. aureus* W1, W3, CICC 21600, C1, and CICC 22942 after cold stress (Figure 3C–G). Meanwhile, the mRNA transcription level of the *icaR* gene in these strains was downregulated or showed no difference. Moreover, the transcription level of the genes *cfla*, *fnbpA*, *sasG*, or *spa* in *S. aureus* W1 and CICC 22942 strains was enhanced over 2-fold (*p* < 0.01). 

Compared with the control sample, the mRNA levels of the *icaA* and *cfla* genes in *S. aureus* CICC 10788 cells after cold stress were increased less than 1.8-fold (*p* < 0.05). There was no difference for the other genes in *S. aureus* CICC 10788 cells (Figure 3H).

Compared with the control group, the transcription levels of the gene *icaA*, *icaB*, and *icaD* gene were decreased over 2-fold (*p* < 0.05) in *S. aureus* CICC 10201 strains after cold stress. The transcription levels of the *icaR, cfla*, and *fnbpA* genes were enhanced 1.8–2.5 folds (*p* < 0.01). The transcription level of the *sasG* or *spa* gene was downregulated.

Compared with the control sample, the mRNA level of the *icaA* or *fnbpA* gene was increased over 1.4-fold (*p* < 0.05) in *S. aureus* C4 cells after cold stress. The transcription level of the *icaC, cflA, sasG*, or *spa* gene was downregulated 1.6–5 fold (*p* < 0.05).

**Figure 4:**
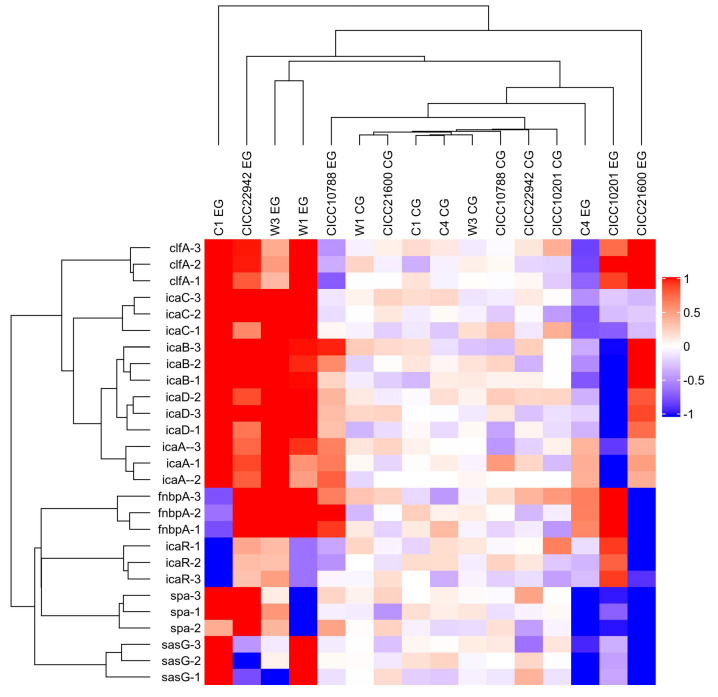
Heat map of the transcription levels of genes in the *S. aureus* biofilm cells under cold stress. Note: *S. aureus* without cold stress was used as the control group. The gene transcription levels in biofilm cells of *S. aureus* after cold stress for 20 weeks at −20 °C were tested. Meanwhile, EG indicated the experimental group, and CE was the control group. The red square indicated that the transcription levels of genes were obviously increased. The blue square indicated significantly decreased gene transcription.

Furthermore, the red square represents the increased gene transcription in the resulting heat map (Figure 4). The color of the control group was lighter, and the value was closer to 0. Obviously, the strains with red plates were seen on the left side. Compared with the control group, the mRNA levels of the *clfA, icaA*, *icaB*, *icaC*, *or icaD* gene were increased in *S. aureus* CICC 22942, W1, W3, and C1 biofilm cells after cold stress for 20 weeks at −20 °C. In addition, the blue square was also viable on the right side. Compared with the control group, the transcription levels of the *icaABCD*, *spa*, or *sasG* gene were decreased in the cells of *S. aureus* CICC 10201 and C4 biofilm after cold stress.

### 3.4. The Polysaccharide and Protein Content in the Biofilm Matrix of S. aureus Cells

**Figure 5:**
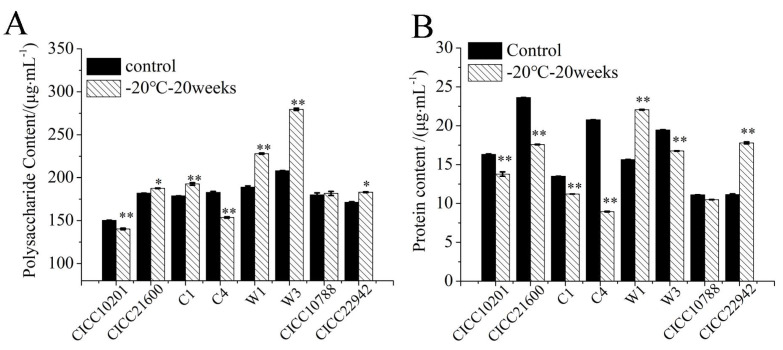
The polysaccharide and protein content in the biofilm matrix of *S. aureus* cells Note: (**A**): The polysaccharide content in the biofilm matrix of *S. aureus* cells. (**B**): The protein content in the biofilm matrix of *S. aureus* cells. *S. aureus* cells without cold stress were used as a control sample. The strains with cold stress were test group. **: Compared with the control group, the tested samples were significantly different (*p* < 0.01), *: Compared with the control group, the treated samples were different (*p* < 0.05). The values were means ± SD (*n* = 3).

The polysaccharide content in biofilms was tested by the phenol sulfate method. *S. aureus* CICC 10201, CICC 21600, C1, C4, W1, W3, CICC 10788, and CICC 22942 with cold stress were the test groups, and strains without cold stress were used as the control samples (Figure 5A). Compared with the control groups, the polysaccharide contents in biofilm matrix of *S. aureus* CICC 10201 and C4 cells after cold stress were significantly decreased (*p* < 0.01). The polysaccharide contents of *S. aureus* CICC 21600, C1, W1, W3, and CICC 22942 after cold stress were significantly increased (*p* < 0.05 or *p* < 0.01). Meanwhile, there was no significant difference in *S. aureus* CICC 10788 cells with or without cold stress. These results were consistent with the quantitative transcription levels of *ica* genes in *S. aureus* strains after cold stress, which was related to the polysaccharide intercellular adhesin (PIA).

A BCA kit was used to quantitatively detect the protein content in the biofilm matrix of *S. aureus* after cold stress (Figure 5B). The strains without cold stress were used as the control groups. Compared to the controls, the protein contents of *S. aureus* W1 and CICC 22942 were significantly increased after 20 weeks of cold stress at −20 °C (*p* < 0.01). However, that of strains CICC 10201, CICC 21600, C1, C4, and W3 after cold stress decreased significantly (*p* < 0.01). There was no difference in *S. aureus* CICC 10788 cells with or without cold stress, and the total protein content was far lower than the polysaccharide content.

### 3.5. Hydrophobicity of S. aureus Strains after Cold Stress

**Figure 6:**
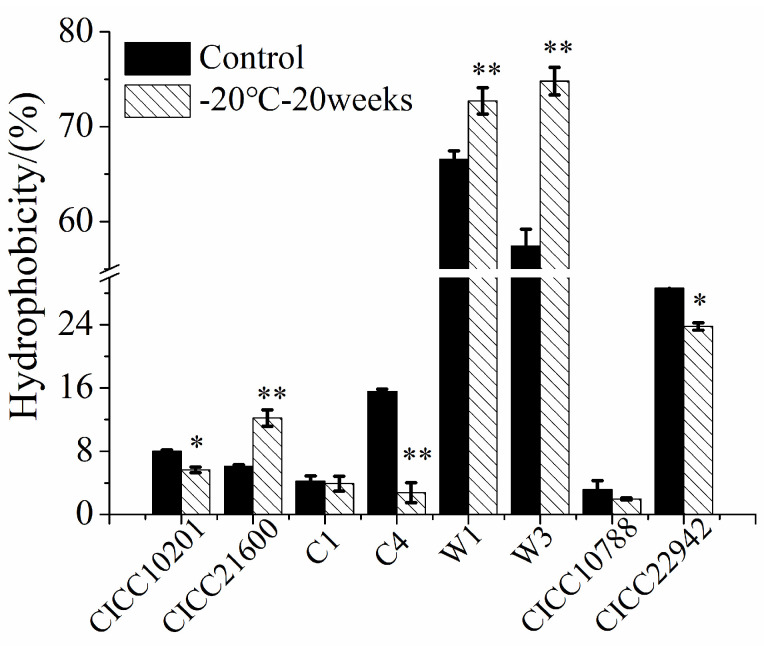
Hydrophobicity of *S. aureus* strains after cold stress. Note: The *S. aureus* CICC 10201, CICC 21600, CICC 22942, CICC 10788, W3, W1, C1 and C4 with cold stress were the test groups, and strains without cold stress were used as the control samples. *: Compared with the control group, the treated samples were different (*p* < 0.05). **: Compared with the control group, the tested sample were significantly different (*p* < 0.01). The values were means ± SD (*n* = 3).

Compared with the control group, the hydrophobicity of *S. aureus* CICC 21600, W1, or W3 cells was significantly increased (*p* < 0.01, Figure 6). The biofilm cells were more difficult to clean up after cold stress at −20 °C for 20 weeks. Compared with the control sample, the hydrophobicity of *S. aureus* CICC 10201, C4, or CICC 22942 cells under cold stress was significantly reduced (*p* < 0.05). Meanwhile, the biofilm cells were easier to purge, which was consistent with the previous experimental results. At the same time, there was similar hydrophobicity of *S. aureus* C1 or CICC 10788 cells with or without cold stress. In addition, the hydrophobicity of *S. aureus* W1 and W3 cells was higher than that of other strains. The biofilm formation ability of *S. aureus* W1 or W3 strain was also stronger than the other strains.

### 3.6. Antibiotics and Enzymes Degraded S. aureus Biofilm

**Figure 7:**
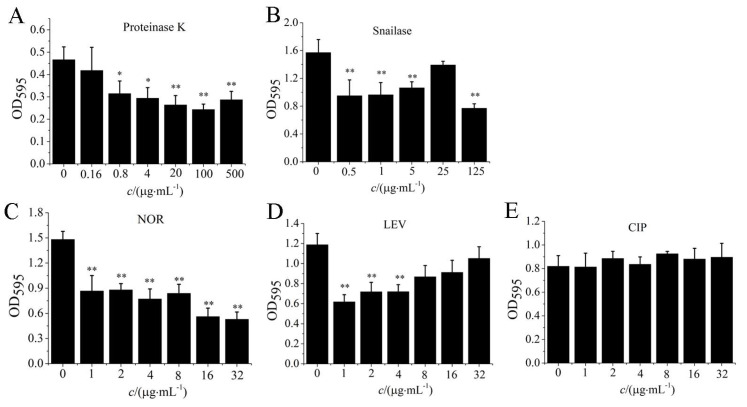
Effect of antibiotics and enzymes on *S. aureus* biofilm. Note. OD_595_ values of crystal violet retention were measured to determine biofilm formation. After treating with proteinase K (**A**), snailase (**B**), norfloxacin (**C**), levofloxacin (**D**) and ciprofloxacin (**E**), the biofilm biomass of *S. aureus* W3 was represented by the OD value at 595 nm. NOR: norfloxacin, LEV: levofloxacin, CIP: ciprofloxacin. The biofilm of *S. aureus* W3 treated with antibiotics or enzymes at a concentration of 0 was used as the control group. “*” means *p* < 0.05, “**” means *p* < 0.01, compared to control. The values were means ± standard deviations (*n* = 6).

Different concentrations of norfloxacin (NOR), levofloxacin (LEV), ciprofloxacin (CIP), proteinase K, and snailase were used to remove biofilm of *S. aureus* W3 cells. When the concentration of proteinase K was higher than 20 µg/mL, the *S. aureus* biofilm could be effectively eliminated (*p* < 0.01, Figure 7A). And the biofilms of *S. aureus* W3 cells could also be effectively degraded by the 125 µg/mL snailase (*p* < 0.01, Figure 7B). In addition, the biofilms of *S. aureus* W3 strains were removed effectively through different concentrations of NOR (*p* < 0.01), and the biofilm content was reduced 62% by the 16 µg/mL concentration of antibiotics NOR (Figure 7C). Similarly, biofilms of *S. aureus* W3 cells could also be removed by antibiotics LEV in the 1–8 µg/mL range of concentration (*p* < 0.05, Figure 7D). The efficiency of LEV to remove biofilm was lower than 47%. By contrast, CIP treatment had no effect on biofilm clearance (Figure 7E). 

### 3.7. The Proteinase K, Snailase, or 84 Disinfectant Degraded S. aureus Biofilm

**Figure 8:**
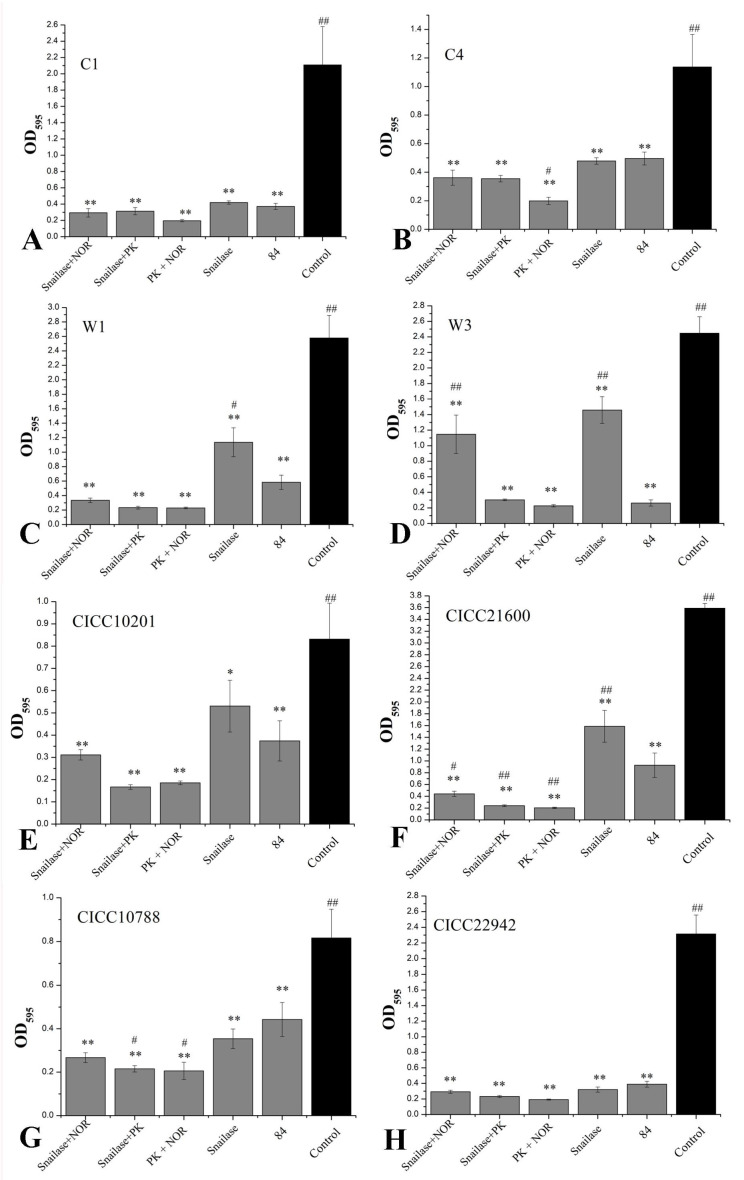
The enzymes mixture degraded *Staphylococcus aureus* biofilm. Note. OD_595_ values of crystal violet retention were measured to determine biofilm formation. The biofilm of *S. aureus* C1 (**A**), *S. aureus* C4 (**B**), *S. aureus* W1 (**C**), *S. aureus* W3 (**D**), *S. aureus* CICC 10201 (**E**), *S. aureus* CICC 21600 (**F**), *S. aureus* CICC 10788 (**G**) and *S. aureus* CICC 22942 (**H**) was degraded by several reagents. PK: proteinase K, NOR: norfloxacin. The untreated biofilm of *S. aureus* was used as the control group. “*” means *p* < 0.05, “**” means *p* < 0.01, compared to control group. “^#^” means *p* < 0.05, “^##^” means *p* < 0.01, compared to 84 disinfectant group. The values were means ± SD (*n* = 6).

The snailase and norfloxacin mixture (125 µg/mL + 16 µg/mL), snailase and proteinase K mixture (125 µg/mL + 20 µg/mL), NOR and proteinase K mixture (16 µg/mL + 20 µg/mL), and snailase (125 µg/mL) and 84 disinfectant (disinfectant with sodium hypochlorite, 1%) were used to remove biofilm of *S. aureus* CICC 10201, CICC 21600, CICC 22942, CICC 10788, W3, W1, C1, or C4 cells. The results showed that the biofilms of *S. aureus* strains were degraded through the mixtures, snailase, or 84 disinfectant (*p* < 0.01, Figure 8). The clearance rate to *S. aureus* C1 and CICC 22942 was higher than 80% and there was no significant difference in experimental groups (Figure 8A,H). Especially compared to the 84 disinfectant group, the norfloxacin and proteinase K mixture could remove the biofilms of *S. aureus* C4 cells more significantly (*p* < 0.05), and the clearance rate was 82% (Figure 8B). Similarly, the clearance rates of the three mixtures to the biofilms of *S. aureus* W1, W3 (except snailase and NOR mixture) or CICC 21600 were higher than 87% (Figure 8C,D,F). By contrast, compared to 84 disinfectant, the proteinase K mixtures treatment had a definite effect on biofilm clearance to *S. aureus* CICC 10201, CICC 21600, and CICC 10788 (Figure 8E–G). In addition, snailase and proteinase K mixture (125 µg/mL + 20 µg/mL), NOR and proteinase K mixture (16 µg/mL + 20 µg/mL) were the most effective in biofilm removal of *S. aureus* cells. The snailase (125 µg/mL) and 84 disinfectant (1%) could also be used to efficient remove the *S. aureus* biofilm in industry.

## 4. Discussion

In the food industry, the contamination caused by *Staphylococcus* biofilm is mainly attributable to three factors: raw food materials, the food processing environment and equipment, and nonstandard operation of staff [31]. Frozen food [32], cooling tubes, freezers [33,34], and operators’ bodies and clothes [35] are easy to ignore. Under appropriate conditions, bacteria choose a specific surface for adherence, thus forming a biofilm. It has been reported that environmental stresses such as temperature, pH, or oxidation can promote the formation of biofilms by pathogenic bacteria [36]. Biofilm formation of *S. aureus* promotes its survival and growth in the food production process, providing more convenient conditions for foodborne disease outbreaks [37]. The formation of biofilms of *S. aureus* cells after cold stress should be considered. This study provided theoretical evidence to efficiently control *S. aureus* in frozen foods.

Eight coagulase-positive *S. aureus* strains were treated with freezing temperature (−20 °C), which is commonly used in the food industry. The biofilm formation of 5 strains of *S. aureus* after cold stress at −20 °C for 20 weeks, including CICC 21600, CICC 22942, W1, W3 and C1, was significantly increased. Therefore, the major matrix that contained polysaccharides and proteins was tested. Many papers have reported that polysaccharide intercellular adhesin (PIA) is an important component of the biofilm matrix. The synthesis of PIA promotes the formation of aggregated biofilms by free cells [13]. PIA is mainly regulated by *ica* gene cluster, which contains the key *icaABCDR* genes. The *icaA*, *icaB*, *icaC*, and *icaD* genes positively regulate PIA, while *icaR* negatively regulates PIA [12,27]. The *icaA* gene encodes N-acetylglucosamine transferase, which catalyzes the synthesis of N-acetylglucosamine into PIA polymer. Furthermore, when both of the *icaA* and *icaD* genes were expressed, the activity of N-acetylglucosamine aminotransferase increased significantly. This means that the product of the *icaD* gene provided the best efficiency for IcaA [13,38]. The *icaC* gene encodes a product that transfers poly-acetylglucosamine to the surface of bacterial cells. The product of the *icaB* gene is the N-deacetylase responsible for partial deacetylation of PIA [39]. PIA is related to the comprehensive biofilm formation of gram-positive and gram-negative bacteria [40]. The transcription of the *icaA* gene cluster is affected by the environmental conditions [11]. Similarly, quantitative PCR results showed that the level of *icaA, icaB, icaC*, *or icaD* genes transcription in *S. aureus* (CICC 21600, CICC 22942, W1, W3, or C1) strains after cold stress was increased > 2-fold (*p* < 0.01). Furthermore, the polysaccharide content in these strains was significantly higher than that of the control (*p* < 0.05, *p* < 0.01). However, the biofilm formation of the *S. aureus* CICC 10201 or C4 strain after cold stress was reduced significantly (*p* < 0.01), and the mRNA level of *ica* gene was decreased more than 2-fold (*p* < 0.05). This meant that the biofilm formation of *S. aureus* cells after cold stress had certain regularity, and the increased transcription of positive regulatory genes of PIA was the main reason for the increase in biofilm formation.

Protein adhesins also play an important role in biofilm formation, especially in the strains lacking the *ica* gene [40]. These adhesion proteins include *S. aureus* surface protein (sasG) [41], clumping factor A (cflA) [42], *Staphylococcus* protein A (spA) [43], and fibronectin-binding protein A (fnbpA) [44]. In the biofilm cells of eight strains of *S. aureus* after cold stress, at least one of the *sasG*, *cflA*, *spa* or *fnbpA* gene transcription was increased more than twofold. The content of biofilm matrix proteins of *S. aureus* W1 or CICC 22942 after cold stress was increased. However, that of *S. aureus* CICC 21600, C1, or W3 strains after cold stress with more biofilm formation tended to decrease. In addition, the protein content in the biofilm matrix of the *S. aureus* strain was far lower than that of polysaccharides. It was speculated that the protein adhesin played an auxiliary role in the biofilm formation of *ica*-positive *S. aureus* strains, which is only one of the reasons for biofilm formation.

Finally, cell surface hydrophobicity (CSH) plays a key role in microbial adhesion by enhancing hydrophobic interactions between cells and biological or abiotic surfaces [29,45,46]. Compared with hydrophilic surfaces, including glass or metals (stainless steel), bacterial cells can adhere to hydrophobic and nonpolar surfaces (plastics) more quickly [46]. It has been reported that hydrophobicity makes microorganisms more virulent. Hydrophobicity promotes the formation of biofilms by enhancing the adhesion of microorganisms, thus improving the antibiotic resistance of cell communities and increasing their virulence [47,48]. Ran reported that one of the reasons for the survival and biofilm formation of *Enterococcus faecalis* under glucose starvation stress was the increase in cell surface hydrophobicity [49]. Angiolella confirmed that 60% of *Malassezia* strains were hydrophobic and adhesive and that biofilm formation was correlated [29]. However, it has also been reported that there is no correlation between hydrophobicity and cell adhesion [50]. In this study, hydrophobicity affected the biofilm formation of *S. aureus* CICC 10201, CICC 21600, C4, W1, or W3 after cold stress, which may be related to the strain specificity.

Due to the complexity of the biofilm matrix, the physical penetration of antimicrobial agents was reduced [51]. In addition, antibiotics may be adsorbed in the matrix and cannot act on microorganisms because of their hydrophilic and anionic properties, making the treatment ineffective [52,53]. Similarly, the use of antibiotics (NOR, LEV, CIP) in this paper against *S. aureus* biofilm was not very effective. Moreover, enzymes were mostly used to remove the biofilm matrix. For example, the biofilm of *Pseudomonas aeruginosa* was more difficult to degrade, and it could be dispersed with deoxyribonuclease I (DNase I) [54]. Treatment of biofilms with DNase I could also improve the effectiveness of antibiotics [55]. Proteases (such as exogenous proteinase K, trypsin, or neutral protease) could effectively degrade part of the biofilm of pathogenic bacteria [56,57]. Proteinase K was also used in the article. Proteinase K was mainly used to degrade the protein components in the biofilm matrix and improve the effectiveness of antibiotics against the *S. aureus* biofilm. Snailase, as a kind of lysozyme, could degrade polysaccharides, such as glucan, chitosan, pectin, etc [58]. In this study, snailase could efficiently remove 8 strains of *S. aureus* biofilms and improve the effectiveness of antibiotics. This method of using snailase or proteinase K provided certain data support for biofilm removal.

Worldwide, contamination by pathogenic bacteria is likely to cause outbreaks even after freezing for 12 to 20 weeks, and food processing equipment should be cleaned in a timely manner for food safety. In addition, *S. aureus* demonstrated strong cold tolerance. Polysaccharides had a major role in its biofilm formation matrix. Adhesion proteins and hydrophobicity also had supporting roles. Proteinase K and snailase can degrade the *S. aureus* biofilm, which provides a way for effective control of *S. aureus*.

## Figures and Tables

**Table 1:** Step OnePlus Realtime PCR system.

Stage	Repeats	Temperature (°C)	Time
Stage1 Initial denaturation	Repeat 1	95	5 min
Stage2 Cyclic reaction	Repeats 40	95	10 s
		60	30 s
Stage3 Dissociation curve	Repeat 1	95	15 s
		60	1 min
		Raise to 95 °C at 0.3 °C/s	15 s

**Table 2:** Primers used in this investigation.

Gene	5’-3’ Nucleotide Sequence	Amplification Product (bp)	Reference	Efficiency (%)
*icaA*	AAGCCAACGCACTCAATCAAGG	151	[27]	73.92
	GGATTACCTGTAACCGCACCAAG			
*icaD*	ACCCAACGCTAAAATCATCG	211	[27]	75.28
	GCGAAAATGCCCATAGTTTC			
*icaR*	TTTTCAGAGAAGGGGTATGACGG	289	This study	72.75
	TTCCAGAAAATTCCTCAGGCGTA			
*icaB*	ATACCGGCGACTGGGTTTAT	176	This study	75.06
	TTGCAAATCGTGGGTATGTGT			
*icaC*	CTTGGGTATTTGCACGCATT	209	[27]	74.34
	GCAATATCATGCCGACACCT			
*cflA*	GCGTGGCTTCAGTGCTTGTA	219	This study	70.08
	CCACACTCGTTTCGCCATTA			
*fnbpA*	CGACACAACCTCAAGACAATAGCGG	146	This study	69.47
	CGTGGCTTACTTTCTGATGCCGTTC			
*spa*	GCTTAAAACCGCAAAATCACGC	143	This study	68.26
	AACCTCAGGCACATTCAAAGC			
*sasG*	AACCTGGTGAAGAGCGAGTG	209	This study	73.81
	GCTCGGCTTCTCTGGGTTTT			
*16S-rRNA*	GGGACCCGCACAAGCGGTGG	191	[27]	99.17
	GGGTTGCGCTCGTTGCGGGA			

## Data Availability

Not applicable.
